# A pilot retrospective study of a physician-directed and genomics-based model for precision lifestyle medicine

**DOI:** 10.3389/fmed.2023.1239737

**Published:** 2023-10-23

**Authors:** Michael Mallin, Jane Hall, Maria Herlihy, Eduard J. Gelman, Michael B. Stone

**Affiliations:** ^1^Wild Health, Inc., Lexington, KY, United States; ^2^Jane Hall Biomed, LLC., Seattle, WA, United States

**Keywords:** precision medicine, genomics, health coaching, chronic disease, diabetes, cardiovascular disease, primary care

## Abstract

Precision lifestyle medicine is a relatively new field in primary care, based on the hypothesis that genetic predispositions influence an individual’s response to specific interventions such as diet, exercise, and prescription medications. Despite the increase in commercially available genomic testing, few studies have investigated effects of a physician-directed program to optimize chronic disease using genomics-based precision medicine. We performed an pilot, observational cohort study to evaluate effects of the Wild Health program, a physician and health coach service offering genomics-based lifestyle and medical interventions, on biomarkers indicative of chronic disease. 871 patients underwent genomic testing, biomarker testing, and ongoing health coaching after initial medical consultation by a physician. Improvements in several clinically relevant out-of-range biomarkers at baseline were identified in a large proportion of patients treated through lifestyle intervention without the use of prescription medication. Notably, normalization of several biomarkers associated with chronic disease occurred in 47.5% (hemoglobin A1c [HbA1c]), 33.3% (low density lipoprotein particle number [LDL-P]), and 33.2% (C-reactive protein [CRP]). However, due to the inherent limitations of our observational study design and use of retrospective data, ongoing work will be crucial for continuing to shed light on the effectiveness of physician-led, genomics-based lifestyle coaching programs. Future studies would benefit from implementing a randomized controlled study design, tracking specific interventions, and evaluating physiological data, such as BMI.

## 1. Introduction

Every individual has different and unique risk factors for the development of chronic diseases such as cardiovascular disease, insulin resistance, and inflammation. This risk is determined by a complex interplay between both modifiable and nonmodifiable factors such as genomics, epigenetics, and lifestyle factors. Although there is significant scientific interest in utilizing these factors in understanding disease processes, to date, few opportunities exist for consumers to engage with healthcare in this method of risk modulation. While the American College of Medical Genetics states “the application of exome and genome sequencing screening tests for apparently healthy individuals is in its infancy,” it also highlights that “the public’s interest in obtaining their own genomic information is likely to increase along with demands on health-care providers to assist patients in accessing testing, interpreting results, and using results in medical care (1).” While there has been considerable interest in expanding health-care provider assistance to the public (2, 3), currently, most consumers engage with direct-to-consumer programs and products which offer genetic and laboratory testing in the absence of a doctor-patient relationship. These user-directed methods fail to provide the benefit of clinically relevant interpretations and subsequent recommendations.

Since the mapping of the human genome in 2008, precision medicine research has skyrocketed with an extraordinary number of new genome wide association studies. Although this has led to ample “clickbait” news articles, very little research has been focused on clinical healthcare delivery. Despite this, there is evidence that genetic predispositions do have a significant effect on clinical response to lifestyle interventions in the general populations (1–3) and, furthermore, that lifestyle interventions can modify risk of disease (4–6). At the heart of this knowledge is the hypothesis that since humans respond differently to specific interventions based on their genetic predispositions, prior knowledge of those predispositions may improve behavior change and thus the success of the interventions. Although this has been preliminarily studied in lifestyle coaching, few studies have evaluated the effect of a lifestyle program implemented by both physicians and health coaches using a multi-omic approach consisting of genomics, epigenetics, laboratory, lifestyle, and microbiome analysis (7).

The Wild Health program encompasses genomic testing, biomarker analysis, physician consultation and health coaching, with a focus on lifestyle intervention to prevent and/or treat common chronic diseases such as pre-diabetes, diabetes, and hyperlipidemia. Physician recommendations for specific lifestyle interventions were discussed with patients, who subsequently received health coaching support through motivational interviewing and structured behavior change. Patients received lIfestyle and behavioral recommendations based on their genetic and laboratory data, as well as their specific goals and pre-existing conditions. Recommendations were comprehensive and covered key pillars of health including nutrition, training, sleep optimization, stress reduction, and supplement use as needed for vitamin or micronutrient deficiencies.

The purpose of this study was to pilot the analytic arm of the Wild Health program, explore trends in patient health during the program, and identify areas of focus for future study (i.e., promising targets for intervention in a more data-rich and controlled study environment). Specifically, we retrospectively examined laboratory biomarkers for patients enrolled with Wild Health over a 2-year time frame resulting in 871 participants with repeat laboratory testing. Our primary goal was to investigate whether participation in the Wild Health program could be associated with improvement of key biomarkers indicative of chronic illness, by comparing pre-intervention values (baseline values at enrollment) vs. post-intervention values (follow-up measurements taken at least 30 days after enrollment). Our secondary goal was to describe pre-intervention and post-intervention values across all biomarkers for which data was available in this cohort.

## 2. Methods

### 2.1. Study setting

Wild Health is a precision lifestyle medicine program that encompasses genomic testing, biomarker analysis, and health coaching for patients across the U.S. provided by a multidisciplinary team of clinicians and health coaches. Patients enter the program most commonly through self-referral and receive follow-up for at least 12 months. Although the number of follow up visits with clinicians and health coaches is variable and based on clinical necessity and patient availability, all patients have an initial consultation including genomic testing, laboratory baseline analysis, and background data collection. All patients included in this study also had at least one additional visit with repeat laboratory testing.

The patient journey is such that after sign-up the patient completes an at-home genetics test and obtains baseline labs at a local blood draw site. The precision medicine and health coaching is then delivered via a series of telemedicine consultations with clinicians and health coaches. Patients start with a 30-min initial coaching consultation to identify current lifestyle patterns and health and wellness goals. Subsequently, a 60-min medical consultation is conducted, including providers and health coaches trained in precision medicine. Wild Health Clarity is a data aggregation tool designed to help physicians interpret large datasets with multiple inputs. The tool uses a combination of single gene and polygenetic analysis combined with patient history and lifestyle as well as laboratory findings to highlight possible disease risk as well as identify more precise lifestyle interventions. This data aggregation allows the care team to interpret complex datasets with efficiency and consistency, consider a larger volume of available evidence regarding patients’ health, and to better inform decision making in cooperation with their patients.

To better demonstrate the design, consider the example of cardiovascular disease risk: Clarity would simultaneously aggregate and display for the clinician the patient’s personal and family history of cardiovascular disease, relevant risk factors, laboratory analysis of lipids, insulin resistance, inflammation, calculated MESA score, genetic cardiovascular risk as assigned by Mega, et al., as well as a genomic-based predisposition to response to a dietary intervention (8–12). The clinician could then use this data to help direct care as they deemed necessary. Similar processes are undertaken for lifestyle pillars including nutrition, exercise, and sleep, and chronic diseases and including dementia, insulin resistance, and inflammation.

Recommended interventions are primarily focused on lifestyle changes including dietary, exercise, sleep, and neurobehavioral interventions. Supplements are recommended when medically appropriate, and prescriptions are rarely used in the event that lifestyle change is deemed unsuccessful or medically necessary. After the initial medical consultation, coaching is provided via telemedicine as needed, with follow up laboratory testing and medical visits with clinicians as clinically necessary.

### 2.2. Study design

The study was a pilot, retrospective observational cohort study of adult patients, aged 18 years and above, who agreed to participate in research and enrolled in the team-based precision medicine program, with index (baseline) visits to Wild Health clinicians between July 2019 and September 2021 for plasma biomarker testing as part of initial health screening. To be included in the study, participants had to have at least one follow-up visit for plasma biomarker collection between 1 and 12 months post-baseline. The study was reviewed and approved by the Institutional Review Board of the Institute of Regenerative and Cellular Medicine. Written informed consent for participation was not required for this study in accordance with the national legislation and the institutional requirements.

### 2.3. Genomic and laboratory testing

After study enrollment, participants were sent a home genomic collection kit which was performed at LabCorp using a specifically designed Illumina SNP chip. The SNP chip used in this study was designed in house on a standard GSA array adding approximately 6,000 additional SNPs to meet requirements supporting multiple validated polygenomic scores along with clinically relevant individual SNPs. Polygenic risk scores were taken from the available clinical literature and include cardiovascular, dementia, exercise, and nutrition, among others (13–17). Individual SNPs were chosen based on literature review and had to meet internal requirements for clinical quality including: (1) the SNP has had consistent and significant odds ratio or positive likelihood ratio with a specific disease process, and (2) has been proven in multiple studies of adequate power and applicability to the patient population.

In addition to the initial laboratory screening panel, epigenetic testing and microbiome testing were performed on an as-needed or desired basis. Initial testing results were entered into a proprietary software program, Wild Health Clarity™ which functions as a data aggregation tool and analyzes genomic, laboratory, lifestyle, biometric, and microbiome data. Biomarker ranges were evaluated via expert review (MD authors) for face validity. No outliers were removed from analysis.

### 2.4. Data analysis

Unadjusted biomarker concentrations at baseline and follow-up were summarized using median and quartile values, along with frequencies and percentages of laboratory values out of range among the cohort. Where multiple repeat measurements were available, one follow-up laboratory value was randomly selected per patient to prevent overcounting patients with more frequent follow up. No tests of hypotheses regarding pre-/post-biomarker measurements were performed at this stage due to lack of *a priori* assumptions and in order to prevent mis-interpretation (Type 1 error) from excessive multiple hypothesis testing. Comparison testing for number of follow up tests in patients in the top quintile of responders to the Wild Health program vs. patients in the bottom quintile was performed via Student’s *t*-test.

For key biomarkers related to chronic illness, all laboratory values, including repeat measurements, were analyzed via covariate-adjusted mixed effects regression modeling, using a pre/post design. The outcome was defined as continuous biomarker at follow up. Model covariates were defined as age, sex, and days since baseline measurement, and pre-intervention (baseline measurement) vs. post-intervention (at least 30 days after baseline measurement). Post-estimation marginal analyses were performed to provide a sample of model-predicted baseline and follow-up laboratory values for illustrative purposes. All analyses were performed in Stata IC 15.1 (College Station, TX).

## 3. Results

### 3.1. Study cohort

During the enrollment period, 2,230 consecutive participants received plasma biomarker testing through Wild Health. Of these, 871 participants had at least one instance of follow-up testing between 1 to 12 months after their baseline test and were included in the study. Repeat testing was performed at the physicians’ discretion and based on medical and clinical utility. The resulting cohort was 49.7% female with a mean age of 48 years (Table 1). Out-of-range [OOR] baseline testing of biomarkers associated with chronic disease demonstrated hyperlipidemia (low density lipoprotein [LDL-C] > 100 mg/dL) in 78.1% of patients tested, diabetes (hemoglobin A1c [HbA1c] ≥ 6.5%) in 30.9% of patients tested, and pre-diabetes (HbA1c 5.7–6.4%) in 9.4% of patients tested. Baseline and follow-up testing generally captured a subset of the 47 available biomarkers with a median of 19 biomarkers (IQR = 5,29). The five most commonly tested biomarkers were Total Cholesterol, LDL-C, HDL-C, Triglycerides, and Protein, and were captured in over 60% of participants (Table 2). At baseline measurement, 826 (94.8%) patients had at least one biomarker out of range (OOR). The median number of OOR biomarkers at baseline was 12 (IQR = 7, 25; Supplementary Figure). The five biomarkers most commonly reported as OOR at baseline were Apolipoprotein B, Homocysteine, Neutrophils, Creatinine, and LDL size.

**Table 1:** Cohort description.

	Frequency (Percent)
*Demographics*	
Age (Mean, SD)	47.6 (12.5)
Male	438 (50.3)
Female	433 (49.7)
Pre-existing Conditions	
*Diabetes Panel*	
High A1C	204 (23.4)
High fasting glucose	137 (15.7)
*Lipids Panel*	
High total Cholesterol	325 (37.3)
Low HDL-C	58 (6.7)
High LDL-C	418 (48)
High Triglycerides	140 (16.1)
*Medications*	
Metformin at baseline	12 (1.4)
Metformin after 30 days	8 (0.9)
Statin at baseline	25 (2.9)
Statin after 30 days	16 (1.8)
*Total Follow-Up Labs*	
1	687 (78.87)
2	140 (16.07)
3+	44 (5.05)

Description of study cohort, *N* = 871.

**Table 2:** Change in out-of-range biomarkers at follow-up.

Biomarker	Total tests	% tested	Baseline OOR		Lab values (Baseline = OOR)		Follow up lab values		Pct. Δ from OOR to in range	
	*N*	Pct	*N*	Pct	Med.	IQR	Med.	IQR	From high	From low
A/G Ratio	474	54.4	72	15.2	2.4	(2.3, 2.6)	2.3	(2.0, 2.5)	40.3	6.9
A1C	427	49.0	204	47.8	9.1	(6.0, 9.6)	5.7	(5.4, 6.1)	47.5	0.0
ALT (SGPT)	459	52.7	256	55.8	29.0	(24.0, 38.0)	26.0	(22.0, 35.0)	19.5	0.0
AST (SGOT)	488	56.0	302	61.9	26.0	(23.0, 33.0)	26.0	(21.0, 32.0)	22.5	0.0
Albumin	481	55.2	30	6.2	5.2	(5.1, 5.3)	4.9	(4.6, 5.0)	66.7	3.3
Alkaline Phosphatase	484	55.6	93	19.2	30.0	(22.0, 37.0)	63.0	(41.0, 93.0)	4.3	60.2
Apolipoprotein B	206	23.7	162	78.6	111.0	(101.0, 135.0)	109.0	(98.0, 126.0)	12.3	0.0
BUN/Creatinine	439	50.4	59	13.4	27.0	(24.0, 34.0)	21.0	(16.0, 24.0)	62.7	5.1
CRP	391	44.9	184	47.1	2.1	(1.3, 4.0)	1.6	(0.9, 3.1)	33.2	0.0
Carbon Dioxide	472	54.2	54	11.4	9.8	(9.5, 19.0)	24.0	(22.0, 25.0)	13.0	74.1
Cholesterol, Total	532	61.1	325	61.1	231.0	(213.0, 254.0)	224.0	(203.0, 247.0)	18.5	2.8
CoQ10	124	14.2	80	64.5	0.6	(0.5, 0.7)	1.0	(0.7, 1.6)	12.5	46.3
Cortisol	178	20.4	104	58.4	9.5	(7.7, 19.7)	10.8	(8.2, 15.9)	13.5	25.0
Creatinine	481	55.2	438	91.1	0.9	(0.8, 1.1)	1.0	(0.8, 1.1)	5.5	0.5
eGFR-Non African American	481	55.2	22	4.6	54.0	(44.0, 56.0)	55.0	(42.0, 60.0)	0.0	27.3
Eosinophils (Absolute)	270	31.0	35	13.0	80.0	(48.0, 129.0)	0.1	(0.1, 0.7)	68.6	0.0
Ferritin/Iron	237	27.2	49	20.7	27.0	(18.0, 509.0)	63.0	(26.0, 312.0)	24.5	22.4
Glucose	521	59.8	137	26.3	107.0	(102.0, 117.0)	100.0	(94.0, 109.0)	49.6	0.0
HDL-C	537	61.7	58	10.8	34.0	(29.0, 36.0)	35.0	(31.0, 42.0)	0.0	37.9
HDL-P	310	35.6	82	26.5	27.9	(24.9, 29.2)	29.2	(25.8, 31.8)	1.2	34.1
Hemoglobin	284	32.6	92	32.4	18.4	(16.3, 42.8)	15.2	(13.7, 16.6)	57.6	7.6
Homocyst(e)ine	408	46.8	386	94.6	9.9	(8.7, 11.6)	9.0	(7.8, 10.3)	16.8	0.0
Insulin	294	33.8	129	43.9	12.2	(9.8, 17.2)	9.0	(6.4, 13.9)	39.5	0.0
LDL Size	336	38.6	310	92.3	21.3	(20.9, 21.5)	21.2	(21.0, 21.5)	0.0	0.3
LDL-C	535	61.4	418	78.1	136.5	(119.0, 159.0)	133.0	(115.0, 156.0)	13.4	0.0
LDL-P	370	42.5	177	47.8	1486.0	(1273.0, 1767.0)	1250.0	(819.0, 1787.0)	33.3	0.0
LP-IR Score	329	37.8	14	4.3	62.0	(50.0, 72.0)	40.0	(27.0, 64.0)	64.3	0.0
Lipoprotein (a)	187	21.5	63	33.7	144.6	(100.2, 211.8)	138.1	(94.8, 205.7)	4.8	0.0
Lp-PLA2 Activity	223	25.6	37	16.6	230.0	(208.0, 241.0)	209.0	(184.0, 235.0)	43.2	0.0
Lymphocytes (Absolute)	270	31.0	66	24.4	4.8	(0.5, 1233.0)	1.7	(1.1, 2.5)	39.4	24.2
MCH	279	32.0	32	11.5	33.1	(24.4, 34.5)	32.2	(25.7, 33.6)	25.0	6.3
Monocytes (%)	267	30.7	104	39.0	10.0	(9.0, 11.0)	9.0	(8.0, 10.0)	34.6	0.0
Neutrophils	248	28.5	203	81.9	57.0	(54.0, 63.0)	58.0	(52.0, 64.0)	8.9	3.0
Omega6: Omega3	272	31.2	39	14.3	14.6	(3.3, 15.9)	8.5	(5.6, 10.8)	41.0	41.0
Omega-3	275	31.6	202	73.5	3.7	(3.1, 4.3)	5.5	(4.3, 7.0)	0.0	53.5
Potassium	479	55.0	57	11.9	44.0	(44.0, 44.0)	4.5	(4.3, 4.7)	87.7	1.8
Protein	540	62.0	144	26.7	10.0	(4.6, 30.0)	7.0	(6.7, 7.3)	45.8	38.2
RBC	285	32.7	131	46.0	845.0	(497.0, 1103.0)	4.9	(4.5, 5.8)	61.8	0.8
Small LDL-P	337	38.7	132	39.2	759.5	(616.5, 970.5)	650.0	(461.0, 885.5)	37.9	0.0
Testosterone	293	33.6	151	51.5	5.3	(2.8, 24.7)	6.6	(2.7, 18.5)	10.6	13.9
TMAO	163	18.7	91	55.8	9.6	(7.4, 17.1)	4.5	(3.3, 9.5)	53.8	0.0
TSH	345	39.6	61	17.7	4.9	(4.1, 6.3)	3.3	(1.8, 4.9)	41.0	9.8
Triglycerides	540	62.0	140	25.9	217.0	(175.0, 260.5)	135.5	(89.0, 212.0)	53.6	0.0
Uric Acid	128	14.7	98	76.6	6.6	(6.0, 8.6)	6.2	(5.6, 7.0)	16.3	0.0
Vitamin B12	226	25.9	123	54.4	427.0	(354.0, 485.0)	701.0	(487.0, 994.0)	8.1	46.3
Vitamin B6	84	9.6	36	42.9	76.8	(56.2, 113.3)	58.5	(31.3, 107.8)	47.2	0.0
Vitamin D	424	48.7	316	74.5	33.5	(27.9, 40.8)	45.9	(37.7, 59.0)	1.3	38.6

“Total Tests” shows total count of tests performed by biomarker, including those with both in range and out-of-range results. “%Tested” shows the percent of the total cohort of 871 participants that were tested for each biomarker. “Baseline OOR” shows the count and percentage of tests that resulted in out-of-range values for each biomarker. “Lab Values (Baseline = OOR)” shows median and interquartile range for baseline lab values, restricted to out of range values. “Follow Up Lab Values” shows median and interquartile range for follow up lab values, restricted to those participants with lab values out of range at baseline. “Pct Δ from OOR to In Range” shows the percentage of participants that shifted from out of range at baseline to in range at follow up, split into those that shifted from high (“From High”) to normal, vs. low to normal values (“From Low”). Biomarkers are sorted alphabetically. OOR, Out of range; Pct, Percent; Med., Median.

### 3.2. A majority of biomarkers out-of-range at baseline demonstrated improvement at follow-up timepoint

To explore changes in biomarkers over time, we focused on the subset of participants and labs that were abnormal at baseline and examined the proportion that shifted to in range. For 34 of the biomarkers tested, 30% or more of participant labs shifted to normal range at a randomly selected follow-up timepoint. For 18 biomarkers, 50% or more participant labs shifted to normal range (Table 2).

### 3.3. Key biomarkers of chronic disease demonstrated improvement at follow-up timepoint

A large percentage of patients demonstrated normalization of key biomarkers associated with metabolic disease at a randomly selected follow-up timepoint, including Hemoglobin A1c (47.5%), fasting glucose (49.6%), LP-IR (64.3%), and fasting insulin (39.5%). Similarly, patients demonstrated normalization of key biomarkers associated with cardiovascular disease including LDL-P (33.3%), LDL-C (13.4%), HDL-C (37.9%), CRP (33.2%), Apo-B (12.3%), Triglycerides (53.6%), and LP-PLA2 (43.2%). We examined medication as a factor and found that improvement at follow-up was not due to metformin or diabetic medication use (in the case of metabolic biomarkers) nor due to statin or other lipoprotein modulating medication use (in the case of cardiovascular biomarkers), with only one patient (or in many cases none of the patients) receiving a new prescription during the treatment period, per biomarker, as noted in Table 1.

### 3.4. Several key biomarkers demonstrated consistent improvement throughout the study period via time- and covariate-adjusted analyses

To continue investigating the biomarkers that were associated with normalization at a single, randomly selected follow-up timepoint in the previous analyses, more in depth analyses were applied. In these analyses, all follow-up laboratory measurements were included in analyses, and time- and demographics-based adjustments were applied, testing for statistically significant differences between pre-intervention (baseline) and post-intervention (at least 30 days post baseline) laboratory values. Based on these models, A1C, insulin, LDL-C, LDL-P, LPIR, and trigylceride levels exhibited significant decreases associated with the intervention. HDL-C exhibited significant increases associated with the intervention (Table 3). For illustrative purposes, we also display the results of post-estimation marginal analysis, which estimates biomarkers values pre- and post-intervention (under the condition that all other covariates are held at their means). This type of model output often allows more intuitive comparisons on the scale of biomarker concentrations, over model coefficients alone.

**Table 3:** Model results from comparison of pre-intervention and post-intervention biomarker levels.

	*N*	Model coefficient (Post- vs. Pre-intervention)	Predicted lab values: pre-intervention	Predicted lab values: post-intervention
A1C	563	−1.9 (−2.2, −1.6)	8.2 (8, 8.4)	6.3 (6.1, 6.5)
Apo B	441	2.1 (−4.6, 8.8)	114.8 (110.4, 119.3)	117 (113.4, 120.6)
CRP	544	0.8 (−2.9, 4.5)	3.7 (1.1, 6.3)	4.5 (2.5, 6.4)
Fasting glucose	400	−18 (−47.1, 11)	141.8 (120.1, 163.5)	123.8 (105.6, 142)
HDL-C	170	5.2 (2.6, 7.8)	31.7 (29.7, 33.7)	36.9 (35.2, 38.6)
Insulin	341	−7.5 (−12.6, −2.5)	19.2 (16, 22.4)	11.7 (8.9, 14.4)
LDL-C	1,142	−6.6 (−11, −2.1)	143.7 (140.4, 147)	137.1 (134.4, 139.8)
LDL-P	523	−242.4 (−391.3, −93.5)	1580.4 (1476.6, 1684.1)	1337.9 (1260.1, 1415.7)
LPIR	49	−17.3 (−29.3, −5.2)	60.4 (52.2, 68.7)	43.2 (36.8, 49.6)
LPPLA2	108	−9.2 (−27.8, 9.4)	223.6 (210.6, 236.5)	214.4 (204.9, 223.9)
Triglycerides	401	−74.6 (−98.7, −50.4)	242.5 (224.6, 260.5)	168 (152.9, 183.1)

Table shows model coefficients for the association of intervention (post vs. pre) with biomarker values, along with 95% confidence intervals. Model predictions of lab values pre- and post-intervention are based on post-estimation marginal analysis (with all other covariate coefficients held at means) and are provided for illustrative purposes.

### 3.5. Spectrum of response in key biomarkers

The highest quintile (top 20%) of responders to the Wild Health program had large reductions from their baseline biomarker measurements. For biomarkers associated with metabolic disease, median Hemoglobin A1c in this group fell by 45.0%, fasting insulin by 72%, glucose by 28%, and LP-IR by 59%, on average. For biomarkers associated with cardiovascular disease, CRP fell by 78%, LDL-P by 76%, Triglycerides by 70%, and HDL-C rose by 44% (Table 4). In contrast, the lowest quintile (bottom 20%) of responders generally had increases from their baseline biomarker measurements (and decrease in HDL-C; Table 4). Patients in the top quintile did not have a significantly different number of follow up tests (Mean = 1.4; 95% CI = 1.3–1.4) than those in the bottom quintile of responders (Mean = 1.3; 95% CI = 1.3–1.4).

**Table 4:** Percent biomarker improvement in highest and lowest quintile of responders.

Biomarker	Highest quintile of improvement	Lowest quintile of improvement
A1C	−44.8 (1.9)	3.1 (12.3)
Apolipoprotein B	−18.6 (7.5)	14 (10.8)
CRP	−78.3 (22.5)	74.8 (85.3)
Glucose	−27.5 (31.9)	5.9 (28.2)
HDL-C	44.2 (127.5)	−15.8 (10.2)
Insulin	−71.6 (30.9)	42 (69)
LDL-C	−26.1 (10.7)	16.7 (13.4)
LDL-P	−75.9 (26.7)	30 (33.5)
LP-IR score	−59.1 (13.5)	−4.3 (4.9)
Lp-PLA2 activity	−24.1 (3.7)	9.6 (12.3)
Triglycerides	−69.6 (5.8)	13.6 (29.5)

Table shows the summary values (Median, IQR) of the top 20% of responders, and the bottom 20% of responders. The top 20% describes greatest decreases in all biomarkers from baseline to follow up, except in the case of HDL-C, where the largest increases were described; and vice versa for the bottom 20%.

## 4. Discussion

We present an analysis of the Wild Health clinical platform consisting of longitudinal biomarker data among participants in the Wild Health program who were exposed to a lifestyle-focused approach encompassing optimization of nutrition, exercise, sleep, stress reduction and supplementation.

While prior research (18–20) has indicated a trend towards normalization of out-of-range biomarkers in patients participating in automated personalized nutrition and lifestyle platforms, to our knowledge, this is the first study evaluating a precision medicine care model (incorporating a physician and health coach team), and demonstrates a trend towards normalization of out-of-range biomarkers indicative of chronic disease.

The effect of the Wild Health program on HbA1c is particularly notable, with substantial improvements as compared to traditional pharmacologic intervention, as well as coaching interventions in the absence of a physician/coach care team (21, 22). Mean HbA1c levels decreased by 1.9% from baseline, as compared to established mean reduction in HbA1c of 1.1% with metformin use (23). Furthermore, 23.0% of patients with initial HbA1c ≥ 6.5% normalized their HbA1c on subsequent testing, effectively inducing remission of diabetes. This is compared to a 3.3% remission rate in those patients receiving standard primary care in the National Diabetes Audit (24).

Significant improvements were noted in patients lipids at follow up, including resolution of elevated LDL-P in 33.3% and resolution of LDL-C in 13.4% of patients. The median reduction in LDL-P was 15.8% and disproportionate to LDL-C reduction of 3.3%. This finding suggests an alteration of LDL particle size and a reduction of small, atherogenic LDL particles consistent with a 19.4% median reduction of small-LDL particles and a 34.7% median reduction in the lipoprotein insulin resistance score from Labcorp (25). Statin therapy has been shown to decrease LDL-P by 30%, suggesting that this precision lifestyle intervention is comparable to statin therapy (26).

Otherwise healthy individuals with elevated C-Reactive Protein (CRP) levels are at up to a 4x higher risk for atherosclerotic cardiovascular disease (27, 28). Lowering of CRP through pharmacologic intervention such as statin therapy is associated with lower incidence of adverse cardiovascular events (29). In our dataset, significant improvements in CRP were noted without the need for statin therapy, including resolution of elevated CRP levels in 33% of patients, suggesting the potential for a marked cardiovascular risk reduction in this patient population. Our results in CRP reduction through lifestyle intervention are similar to the CANTOS trial using 150 mg canakinumab which noted a 17% overall reduction in the risk of MACE (30).

Importantly, the non-randomized observational nature of this study introduced multiple sources of bias that would tend to overestimate the effect size from our study sample if attempting to extrapolate to the general population. “Sufficient data bias” encompasses several biases, and refers to including only patients who have sufficient data for analysis in the study (31). Here, only patients with at least one follow up biomarker measurement were analyzed, which may indicate a more dedicated clinician, a sicker patient, or a more adherent patient, all of which would bias toward positive results; although we did not find that patients in the top quintile of responders had significantly more follow up measurements than those in the bottom quintile. “Healthy user bias” refers to the likelihood that patients that enrolled in the program, or adhered to the program long enough for one follow up biomarker measurement, may have other healthy habits or socioeconomic resources that contributed to their successful normalization of biomarkers associated with chronic disease (32). “Spectrum bias” refers to a focused sampling bias, which precludes the description of intervention effect on the full spectrum of patients with different severity of illness (33). In this study, intentionally focused on patients with out of range biomarkers at baseline for two reasons. Clinically, these are the patients that would be targeted by the Wild Health program for meaningful health interventions. And analytically, this group could be more sensitive to intervention, and allow us to generate hypotheses for follow up in more rigorous future studies. The current study is not meant to show definitive evidence of improvement in biomarkers due to the Wild Health program across all-comers in the population.

We also note that not all patients responded equally to the Wild Health Program. At the time of this study, our ability to delve into the reasons for these differences are limited due to the incomplete nature of our retrospective data source. Specific lifestyle intervention and medical recommendations were not analyzed, and therefore we are unable at this time to identify the level of impact associated with any specific intervention, as opposed to the overall approach provided by the Wild Health program. In addition, adherence to interventions was not tracked.

While this observational study was not designed to evaluate the economic impact of lifestyle-based precision medicine, a discussion of the potential health savings impact is warranted given the costs associated with the treatment of chronic disease. According to the American Diabetes Association, the cost of diabetes in the United States in 2017 was 327 billion dollars with annual direct costs of $9,600 per patient (34). When extrapolated to our patient population in this dataset, of which 30.9% patients met criteria for diabetes on initial labs, the 98 patients with normalization of HbA1c on follow up labs has the potential to save $940,800 dollars annually. Further research is necessary to establish the total cost savings associated with this method of care delivery.

Similarly to diabetes, cardiovascular disease represents a significant cost burden to the American economy with costs estimated as high as 229 billion in 2017 (35). Prior research has found that statin based reduction of LDL cholesterol to normal levels could account for a total annual cost savings of 430 million or $9,900 per patient in 2011 (36). Given that we have shown LDL normalization in 33.3% of patients, comparable as those seen with statins, we would expect an even greater cost savings given the lack of need for statin-based prescription therapy and the associated cost.

## Data availability statement

The raw data supporting the conclusions of this article will be made available by the authors, without undue reservation.

## Ethics statement

The studies involving humans were approved by Institutional Review Board of the Institute of Regenerative and Cellular Medicine. The studies were conducted in accordance with the local legislation and institutional requirements. The ethics committee/institutional review board waived the requirement of written informed consent for participation from the participants or the participants’ legal guardians/next of kin because the studies used de-identified medical records data with no more than minimal risk to the subjects.

## Author contributions

MM and JH conceived the work and wrote the main manuscript text. EG and JH acquired and analyzed the data. JH prepared Tables 1–4. All authors reviewed the manuscript.
