# Supplementary material for: A pilot retrospective study of a physician-directed and genomics-based model for precision lifestyle medicine
Source: Front Med (Lausanne). 2023 Oct 23;10:1239737. doi: 10.3389/fmed.2023.1239737 (PMC10629614; doi:10.3389/fmed.2023.1239737)
Supplement: Supplementary file 1 [file Image_1.pdf]

A pilot retrospective study of a physician-directed and genomics-based model for precision lifestyle medicine

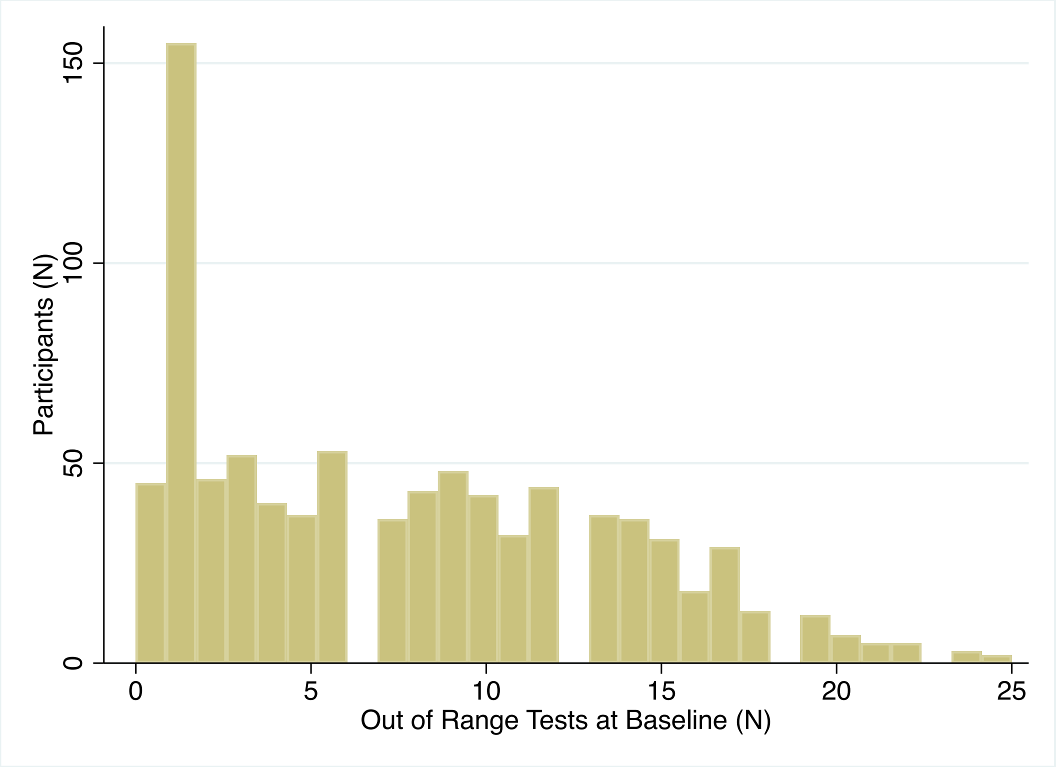

Supplementary Figure. Out-of-range biomarkers at baseline, by participant. Histogram shows counts of tests and participants. Each participant was tested for a subset of the 47 biomarkers available for analysis (median = 19, IQR = 5,29), based on physician guidance.
